# Genetic Analysis of Children With Unexplained Developmental Delay and/or Intellectual Disability by Whole-Exome Sequencing

**DOI:** 10.3389/fgene.2021.738561

**Published:** 2021-11-10

**Authors:** Jingjing Xiang, Yang Ding, Fei Yang, Ang Gao, Wei Zhang, Hui Tang, Jun Mao, Quanze He, Qin Zhang, Ting Wang

**Affiliations:** ^1^ Center for Reproduction and Genetics, The Affiliated Suzhou Hospital of Nanjing Medical University, Suzhou, China; ^2^ Center for Reproduction and Genetics, Suzhou Municipal Hospital, Suzhou, China

**Keywords:** whole-exome sequencing, developmental delay, intellectual disability, exome-based CNV analysis, variants

## Abstract

**Background:** Whole-exome sequencing (WES) has been recommended as a first-tier clinical diagnostic test for individuals with neurodevelopmental disorders (NDDs). We aimed to identify the genetic causes of 17 children with developmental delay (DD) and/or intellectual disability (ID).

**Methods:** WES and exome-based copy number variation (CNV) analysis were performed for 17 patients with unexplained DD/ID.

**Results:** Single-nucleotide variant (SNV)/small insertion or deletion (Indel) analysis and exome-based CNV calling yielded an overall diagnostic rate of 58.8% (10/17), of which diagnostic SNVs/Indels accounted for 41.2% (7/17) and diagnostic CNVs accounted for 17.6% (3/17).

**Conclusion:** Our findings expand the known mutation spectrum of genes related to DD/ID and indicate that exome-based CNV analysis could improve the diagnostic yield of patients with DD/ID.

## Background

Developmental delay (DD) and intellectual disability (ID) are major manifestations of neurodevelopmental disorders (NDDs) with a global prevalence of 1%–3% (Thapar et al., 2017; Ismail and Shapiro, 2019). DD/ID shows phenotypic pleiotropy, and the underlying cause of DD/ID is heterogeneous, in which genetic factors such as copy number variations (CNVs) and variants in single genes have been recognized as major reasons (Savatt and Myers, 2021). With the advent of next-generation sequencing (NGS), the field of genetics was transformed; and the number of genes known to be associated with DD/ID has increased significantly. For example, over 700 genes have been identified in X-linked, autosomal-dominant, and autosomal-recessive ID until 2015 (Vissers et al., 2016).

Chromosomal microarray has been recommended as a first-tier clinical test to identify chromosomal CNVs and regions of homozygosity in individuals with DD/ID, autism spectrum disorders (ASDs), or multiple congenital anomalies with a diagnostic yield of 15%–20% (Manning et al., 2010; Miller et al., 2010). Whole-exome sequencing (WES) could detect single-nucleotide variants (SNVs) and small insertions or deletions (Indels) by whole-exome capture and massively parallel DNA sequencing, which has a diagnostic advantage in situations of genetic heterogeneity or unknown causal genes compared with conventional tests of single gene or gene panels (Monroe et al., 2016). WES is also recommended as a first-tier clinical diagnostic test for individuals with NDDs with an overall diagnostic yield of 36%, including 31% for isolated NDD, and 53% for NDD plus associated conditions, which is greater than the diagnostic yield of CMA (15%–20%) (Srivastava et al., 2019). Recently, CNV calling has been performed by depth-of-sequence coverage analysis of WES data, which enables the detection of deletions and duplications at the exon level (Marchuk et al., 2018).

In this study, WES and exome-based CNV analysis were performed for 17 children with unexplained DD/ID, and a variety of diagnostic variants including SNVs/Indels and CNVs were identified, indicating that WES could help to identify their molecular etiology and the incorporation of CNV calling could improve the diagnostic rate.

## Methods

### Patients

This study was approved by the institutional ethics committee of the Affiliated Suzhou Hospital of Nanjing Medical University. Written informed consent was obtained from each patient’s parents. This study included 17 children with unexplained DD/ID referred to our center for reproduction and genetics, the affiliated Suzhou Hospital of Nanjing Medical University, Suzhou, Jiangsu, China, from January 2018 to March 2021. The characteristics of each patient including age, gender, and main phenotypes are listed in Table 1, and their clinical details are listed in Supplementary Table S1. Global DD is defined as a delay in two or more developmental domains of cognition, speech/language, gross/fine motor, social/personal, and activities of daily living (Mithyantha et al., 2017). Severity of ID was determined based on the intelligence quotient (IQ): severe ID (IQ < 40), moderate ID (IQ range 40–60), and mild ID (IQ range 60–70). In the absence of IQ, ID was diagnosed by the pediatric neurologist or geneticist.

**TABLE 1:** Summary of patients’ clinical manifestations and molecular diagnoses.

Patient ID/sex/age	Phenotype	Gene/locus	Diagnosis (OMIM phenotype)	Variants	Zygosity	Variant type	Inheritance	Classification (ACMG)	References (PMID)
P1/male/2 years	Global developmental delay, intellectual disability	*MED13L*	Developmental delay and distinctive facial features with or without cardiac defects (#616789)	NM_015335.4:c.1284_1,285 insTTTAAGCTTTT (p.Lys429Phefs*7)	Heterozygous	Frameshift	AD; *de novo*	P	—
P2/male/8 years	Perinatal polyhydramnios, intellectual disability, global developmental delay, ataxia, anemia	—	—	—	—	—	—	—	—
P3/male/5 years	Spastic cerebral palsy, febrile seizure, global developmental delay, mild intellectual disability, muscular hypertonia	—	—	—	—	—	—	—	—
P4/male/6 years	Bilateral dislocation of hip joints, clubfoot, strabismus, global developmental delay, intellectual disability	—	—	—	—	—	—	—	—
P5/male/6 years	Cerebral atrophy, seizure, global developmental delay, intellectual disability, muscular hypertonia	*CNPY3*	Developmental and epileptic encephalopathy 60 (#617929)	NM_006586.4: c.283C > G (p.Arg95Gly); c.834del (p.Ter279Gluext*8)	Compound heterozygous	Missense, frameshift	AR maternally inherited, paternally inherited	VUS, VUS	—
P6/female/9 years	Intellectual disability, global developmental delay, hypermetropia	—	—	—	—	—	—	—	—
P7/male/3 years	Global developmental delay, intellectual disability	*SCN2A*	Developmental and epileptic encephalopathy 11 (#613721); episodic ataxia, type 9 (#618924); seizures, benign familial infantile, 3 (#607745)	NM_021007.3:c.1117del (p.Ala373Profs*9)	Heterozygous	Deletion	AD; *de novo*	P	—
P8/male/4 years	Global developmental delay, agenesis of the corpus callosum, low anterior hairline, strabismus	*ARID1B*	Coffin-Siris syndrome 1 (#135900)	NM_001374828.1:c.4194T > G (p.Tyr1398*)	Heterozygous	Nonsense	AD; *de novo*	P	—
P9/male/6 years	Intellectual disability, seizure, autism, cerebral white matter atrophy	*PRRT2*	Convulsions, familial infantile, with paroxysmal choreoathetosis (#602066); episodic kinesigenic dyskinesia 1 (#128200); seizures, benign familial infantile, 2 (#605751)	NM_145,239.2:c.649dup (p.Arg217Profs*8)	Heterozygous	Insertion	AD; maternally inherited	P	22101681, 22870186, 22877996, 25667652, 22243967
P10/female/5 years	Tetralogy of Fallot, autism, global developmental delay	7q11.23	Chromosome 7q11.23 duplication syndrome (#609757)	chr7:72649202-74191713dup, 1.54 Mb	Heterozygous	CNV	*De novo*	P	19249392, 26333794, 19752158
P11/male/9 years	Moderate intellectual disability, global developmental delay	—	—	—	—	—	—	—	—
P12/male/7 years	Mild intellectual disability, delayed speech and language development, low-set ears, downslanting palpebral fissures, short penis	*SETBP1*	Developmental delay, autosomal dominant 29 (#616078); Schinzel-Giedion midface retraction syndrome (#269150)	NM_015559.3: c.2311dup (p.Ser771Phefs*26)	Heterozygous	Frameshift	AD; unknown	LP	—
P13/male/2 years	Premature birth, delayed speech and language development	—	—	—	—	—	—	—	—
P14/male/7 years	Intellectual disability, global developmental delay	*GRIN2B*	Intellectual developmental disorder, autosomal dominant 6, with or without seizures (#613970); Developmental and epileptic encephalopathy 27 (#616139)	NM_000834.4:c.1711del (p.Ala571Profs*80)	Heterozygous	Deletion	AD; *de novo*	P	—
P15/male/7 years	Prenatal hydrocephalus, global developmental delay, mild intellectual disability, retrognathia, strabismus	8p21.2p12	—	chr8:25184869-31641821del, 6.46 Mb	Heterozygous	CNV	*De novo*	P	—
P16/male/6 years	Global developmental delay, moderate intellectual disability, neonatal feeding difficulties, strabismus	19p13.2	—	chr19:1,3044343-13227605del, 183.3 kb	Heterozygous	CNV	*De novo*	P	—
P17/male/4 years	Neonatal asphyxia, global developmental delay, muscular hypertonia	—	—	—	—	—	—	—	—

Abbreviations: CNV, copy number variation; AD, autosomal dominant; AR, autosomal recessive; P, pathogenic; LP, likely pathogenic; VUS, variant of uncertain significance.

### Whole-Exome Sequencing and Data Analysis

Genomic DNA was extracted from the whole blood of the patients and their parents. WES was performed using the SureSelect Human All Exon Kit (Agilent, Santa Clara, CA, USA) and Illumina NovaSeq 6,000 platform (Illumina, San Diego, CA, USA). The sequencing reads were aligned to the human reference genome (hg19/GRCh37) using Burrows-Wheeler Aligner tool, and PCR duplicates were removed by Picard v1.57 (http://picard.sourceforge.net/). The fraction of target bases covered at least 20× should be over 96%, with an average sequencing depth on target bases of over 100×. GATK (https://software.broadinstitute.org/gatk/) was employed for identifying the SNVs and Indels. Variant annotation and interpretation were conducted by ANNOVAR (Wang et al., 2010). The variants were searched in the dbSNP (http://www.ncbi.nlm.nih.gov/SNP/), 1000 Genomes Project database (http://www.1000genomes.org/), Exome Aggregation Consortium (ExAC) (http://exac.broadinstitute.org/), and the Genome Aggregation database (gnomAD) (http://gnomad.broadinstitute.org/). The pathogenicity of mutations was predicted by PROVEAN (http://provean.jcvi.org), PolyPhen-2 (http://genetics.bwh.harvard.edu/pph2/), and MutationTaster (http://www.mutationtaster.org/). Disease and phenotype databases and published literature, such as OMIM (http://www.omim.org), ClinVar (http://www.ncbi.nlm.nih.gov/clinvar), HGMD (http://www.hgmd.org), and PubMed (http://www.ncbi.nlm.nih.gov/pubmed), were also searched. The variants were classified according to the Standards and Guidelines for the Interpretation of Sequence Variants released by the American College of Medical Genetics and Genomics (ACMG) and the Association for Molecular Pathology (Richards et al., 2015). Finally, the variants with minor allele frequency <0.05 were selected for further interpretation considering ACMG category, evidence of pathogenicity, clinical synopsis, and inheritance mode of associated disease.

### Sanger Sequencing

To validate the WES results, the identified candidate variants were amplified by PCR using genomic DNA from the patients and their parents. The primers used for PCR are listed in Supplementary Table S2. The PCR products were purified and sequenced in two orientations using an ABI 3500 Genetic Analyzer (Applied Biosystems, Foster City, CA, USA). The mutation sites were analyzed by comparison with the GenBank reference sequences of each candidate gene.

### Exome-Based CNV Detection and Validation

A comprehensive tool was used for CNV calling. It included XHMM (http://atgu.mgh.harvard.edu/xhmm) and PCA method to remove sequencing noise and CNVKit (https://github.com/etal/cnvkit) fix module to perform GC and bias correction; and then copy number calculation and CNV identification were performed in exons and long segment areas. The identified CNVs were interpreted according to the standards and guidelines for interpretation and reporting of postnatal constitutional CNVs released by the ACMG and the technical standards for the interpretation and reporting of constitutional copy-number variants recommended by the ACMG and the Clinical Genome Resource (ClinGen) (Kearney et al., 2011; Riggs et al., 2020). CNVs were further validated by multiplex ligation-dependent probe amplification (MLPA) or quantitative PCR (qPCR) or CNV sequencing (CNV-seq).

## Results

A total of 17 children with DD/ID were enrolled and analyzed by WES. As shown in Table 1, the age of 17 probands in this study ranged from 2 to 9 years, with a mean age of 5.6 years. Male-to-female ratio was 15:2. The clinical characteristics of 17 probands include global DD (14/17) and ID (13/17) (Table 1, Supplementary Table S1).

### Diagnostic Yields

Among the 17 probands, WES in family trios (Trio-WES) was performed for 14 patients (patients 1–11 and 15–17). Patient 12 and his father underwent WES as a father–proband duo, for the sample of the patient’s mother was unavailable. And patients 13 and 14 had singleton WES. Analysis of WES data revealed that the coverage for over 97% of the targeted bases were over 20×, with an average sequencing depth of over 100×. An overall diagnostic rate of 58.8% (10/17) was achieved after analysis of SNV/Indel and CNV, of which diagnostic SNVs/Indels accounted for 41.2% (7/17) and diagnostic CNVs accounted for 17.6% (3/17) (Table 1).

**FIGURE 1:**
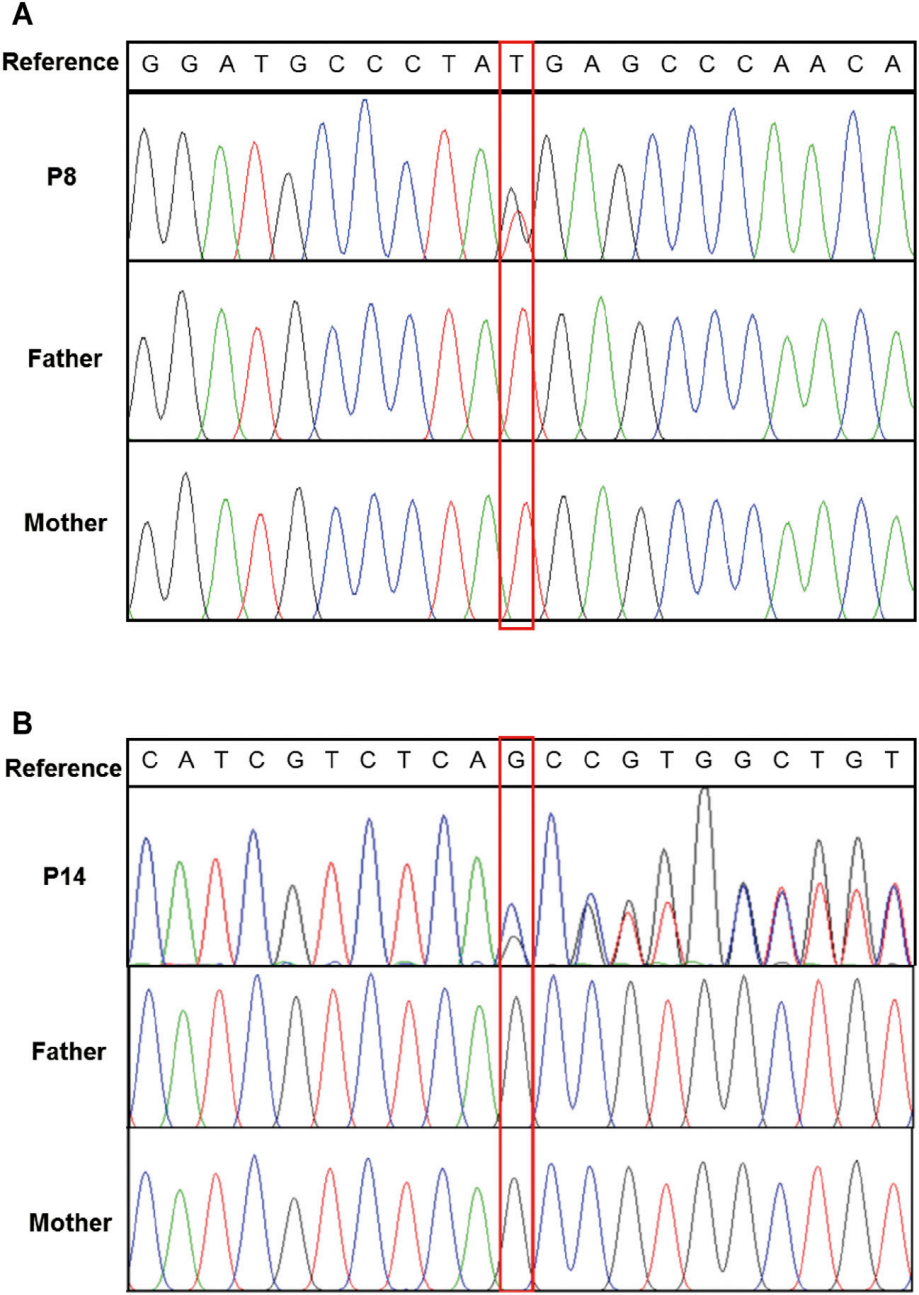
Confirmation of *de novo* variants by Sanger sequencing. **(A)** Sanger sequencing of *ARID1B* in patient 8 and his parents. **(B)** Sanger sequencing of *GRIN2B* in patient 14 and his parents. Variants were indicated by red boxes.

A total of eight variants in seven genes were identified in seven probands by SNV/Indel analysis and confirmed by Sanger sequencing, including six variants in six genes associated with autosomal dominant disorders and two compound heterozygous variants in *CNPY3* gene related to an autosomal recessive disorder early infantile epileptic encephalopathy 60 (EIEE60). In autosomal dominant disorders, four variants detected in patients 1, 7, 8, and 14 occurred *de novo* (Figure 1); the origin of a variant in *SETBP1* gene of patient 12 is unknown, for his mother’s sample is unavailable; and a variant in *PRRT2* gene of patient 9 was inherited from his unaffected mother. The two compound heterozygous variants in *CNPY3* gene of patient 5 were inherited from his mother and father (Table 1).

**FIGURE 2:**
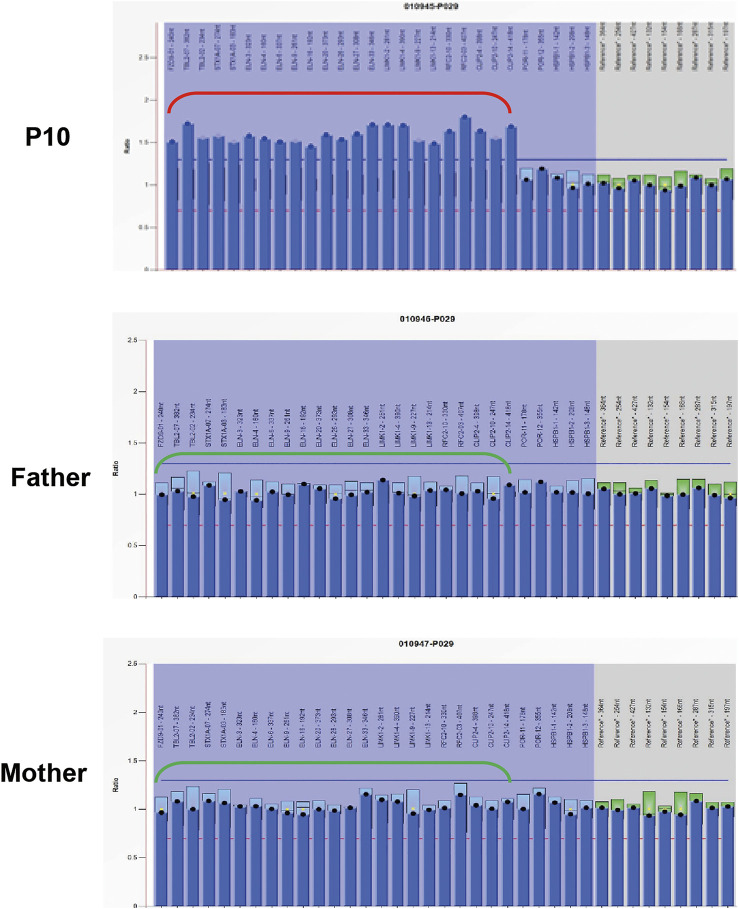
MLPA results of patient 10 and her parents. MLPA was performed using SALSA MLPA Probemix P029 WBS kit, and the red bracket indicates the duplication region in patient 10.

**FIGURE 3:**
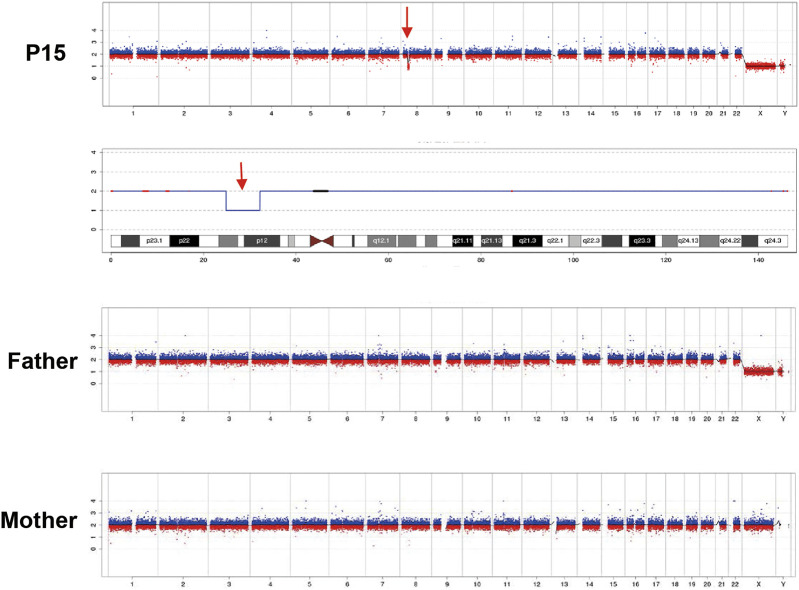
CNV-seq results of patient 15 and his parents. A 6.46-Mb deletion on chromosome 8 of patient 15 is indicated by the red arrow.

**FIGURE 4:**
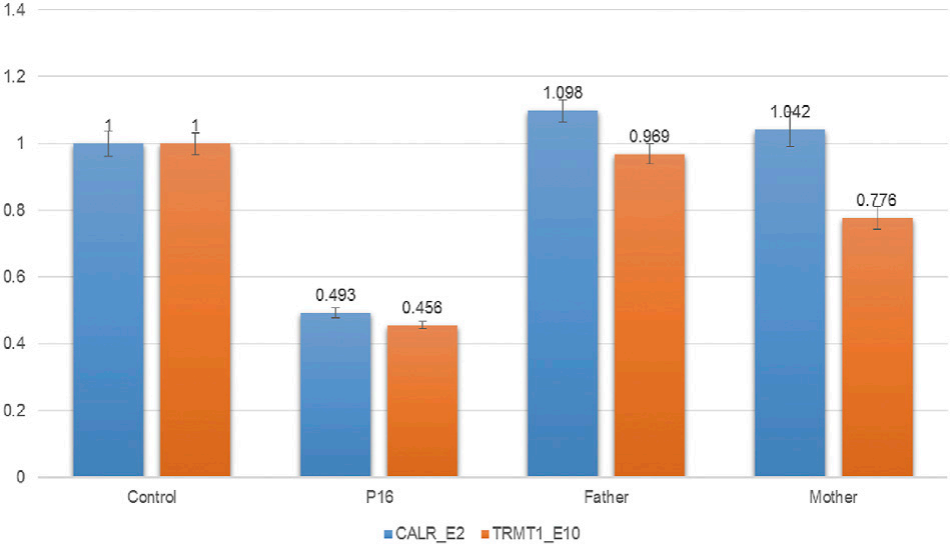
qPCR results of patient 16 and his parents. To confirm the 183.3-kb deletion on chromosome 19p13.2, two pairs of primers for *CALR*_E2 and *TRMT1*_E10 were selected and used in the qPCR. Three replicates were performed, and the relative copy number was estimated by the comparative 2^−ΔΔCT^ method using *ALB* gene as the internal control. The numbers in the *Y*-axis indicate the 2^−ΔΔCT^ value. 2^−ΔΔCT^ < 0.1 is considered to be a homozygous deletion, 0.3 < 2^−ΔΔCT^ < 0.7 is considered to be a heterozygous deletion, 0.7 < 2^−ΔΔCT^ < 1.3 is considered to be normal, and 2^−ΔΔCT^ > 1.3 is considered to be a duplication.

Exome-based CNV analysis revealed three *de novo* pathogenic CNVs. A 1.54-Mb duplication on chromosome 7q11.23 related to chromosome 7q11.23 duplication syndrome (MIM #609757) was identified in patient 10 and confirmed by MLPA (Figure 2). In patient 15, a 6.46-Mb deletion on chromosome 8p21.2p12 was detected and validated by CNV-seq (Figure 3). A 183.3-kb deletion on chromosome 19p13.2 was found in patient 16 and verified by qPCR (Figure 4).

### Case Example

**FIGURE 5:**
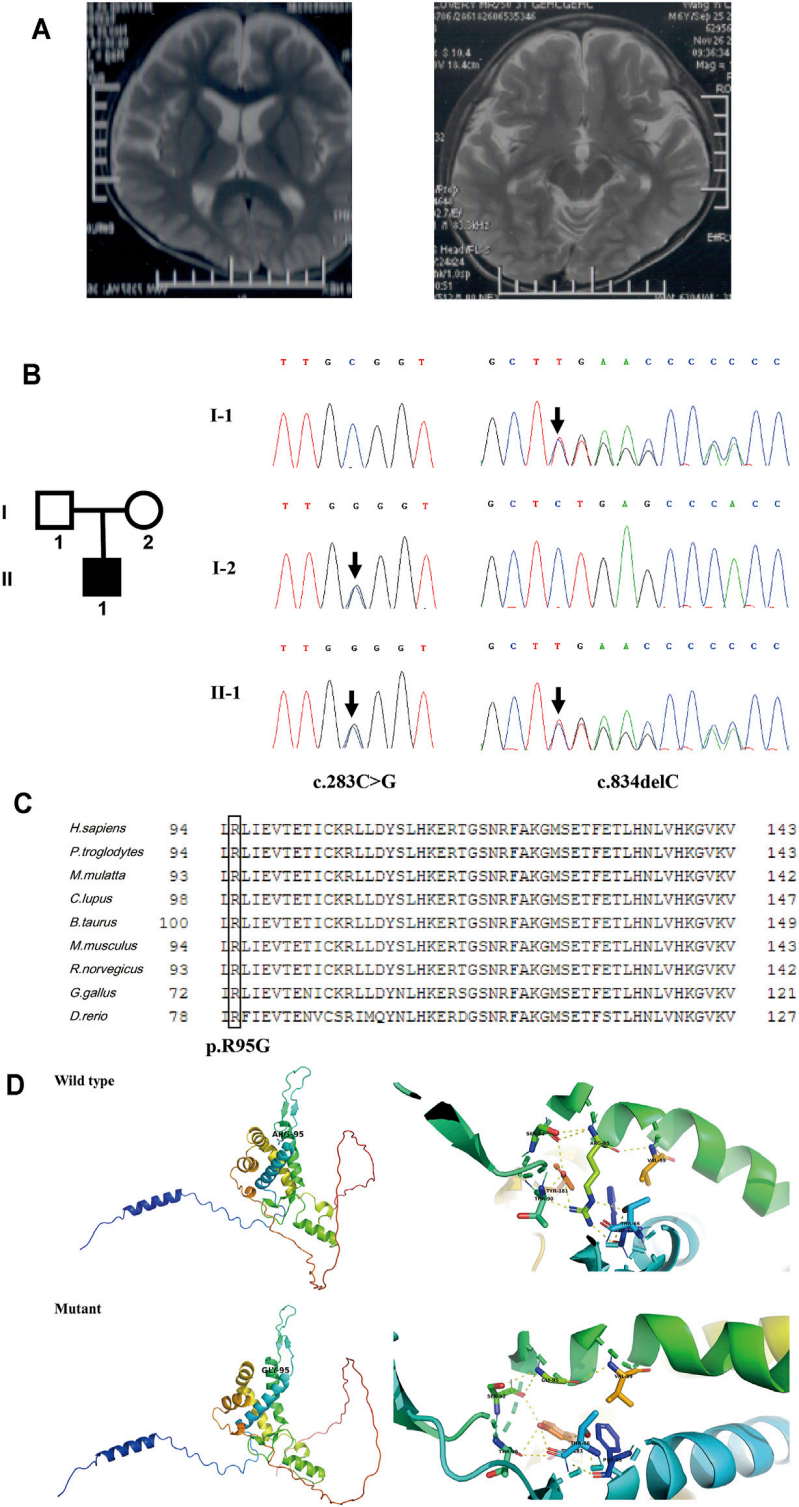
Clinical information and genetic analysis of patient 5. **(A)** Brain MRI of the patient. The left panel shows enlarged lateral ventricles, and the right panel shows bilateral hippocampus. **(B)** Genetic analysis of the family. Left panel: the pedigree of the patient’s family. Right panel: Sanger sequencing of *CNPY3* in family members demonstrated mutations in the proband and his parents. Mutations are indicated by black arrows. **(C)** Multiple sequence alignment of CNPY3 protein and its orthologs in different species. The arginine residue in position 95 is indicated by a black box. The protein and its orthologs were aligned using Clustal Omega (http://www.clustal.org/omega/). **(D)** Predicted structures of wild-type and mutated CNPY3 proteins using PyMOL (http://www.pymol.org). ARG-95 of wild-type protein may form hydrogen bonds with PHE-63, THR-66, THR-90, SER-92, VAL-99, and TYR-181, while GLY-95 of mutant protein may only form hydrogen bonds with SER-92 and VAL-99, which may affect the protein structure and function.

Patient 5 is a 6-year-old boy whose parents are healthy and non-consanguineous. He was born full term by natural delivery without abnormalities, weighing 2,900 g. He was bed ridden and presented with global DD, spastic quadriplegia, ID, and intractable seizure. He had multiple admissions for pneumonia and seizure. His first seizure started at age 6 months, and his electroencephalography (EEG) at 2 years of age showed diffuse sharp waves, spike waves, and multiple spike and slow wave complex. MRI of his brain at 6 years old revealed enlargement of the lateral ventricles and cerebral atrophy (Figure 5A).

WES in family trios (Trio-WES) was performed with DNA from patient 5 and his parents. For patient 5, the coverage for 97.87% of the targeted bases was over 20×. Ultimately, two compound heterozygous variants in *CNPY3* gene (c.283C > G and c.834del) were detected. Sanger sequencing validated the two compound heterozygous variants in the patient. His mother was heterozygous for the c.283C > G variant in exon 3 of *CNPY3* gene, and his father was heterozygous for the c.834del variant in exon six of *CNPY3* gene (Figure 5B). These two variants were not recorded in the dbSNP, 1000 Genomes Project database, ExAC, or gnomAD. The c.283C > G variant was a novel missense mutation causing a substitution from arginine to glycine of CNPY3 protein (p.Arg95Gly), which is predicted to be deleterious by PROVEAN with a score of −6.62, probably damaging by PolyPhen-2 with a score of 1.0, and disease causing by MutationTaster with a probability value of 0.999. Furthermore, sequence alignment of human CNPY3 protein and its othologs in different species revealed that the R95 residue is highly conserved among species (Figure 5C). And protein structural analysis revealed that substitution from arginine to glycine of CNPY3 protein at position 95 may reduce the formation of hydrogen bonds, thus affecting the protein structure and function (Figure 5D). The c.834del variant is a novel frameshift deletion mutation, which results in a prolonged protein with the addition of eight amino acid residues (EPTQHPLS) to the C-terminal of CNPY3 protein (p.Ter279Gluext*8). According to the ACMG variant classification guideline (Richards et al., 2015), the c.283C > G variant could be classified as uncertain significance with two supporting (PM2_Supporting and PP3) evidences, and the c.834del variant could be classified as uncertain significance with one moderate (PM4) and one supporting (PM2_Supporting) evidences.

## Discussion

In this study, WES was performed for 17 probands with DD/ID with an overall diagnostic yield of 58.8% (10/17), which is consistent with a recent study that WES identified pathogenic variants in 53.5% (54/101) of patients with DD/ID (Hiraide et al., 2021). A total of eight *de novo* variants were detected, including five SNVs/Indels and three CNVs, corroborating the burden of *de novo* variants in DD/ID (Brunet et al., 2021). And further functional studies are needed to elucidate the effect of the identified variants at the transcriptional or translational level. In addition, exome-based CNV analysis revealed three pathogenic CNVs, increasing the diagnostic yield by 17.6% (3/17), which is consistent with a recent study that incorporation of exome-based CNV calling improved the diagnostic rate of trio-WES by 18.92% (14/74) in patients with NDDs (Zhai et al., 2021). However, WES analysis yielded negative results for seven patients in this study, possibly due to technical limitations of WES (e.g., deep intron mutations and structural variants).

SNV/Indel analysis identified eight variants of seven genes, including five novel heterozygous variants in patients 1, 7, 8, 12, and 14; one reported heterozygous variant in patient 9; and two compound heterozygous variants in patient 5. A novel *de novo* heterozygous variant c.1284_1285insTTTAAGCTTTT of *MED13L* gene was detected in patient 1, resulting in a frameshift and a premature stop codon (p.Lys429Phefs*7). The *mediator complex subunit 13-like* (*MED13L*) gene encodes a subunit of the mediator complex that functions as transcriptional regulation by physically linking DNA-binding transcription factors and RNA polymerase II in early development of the heart and brain (Utami et al., 2014). *MED13L* haploinsufficiency is involved in DD and distinctive facial features with or without cardiac defects (MIM #616789). In addition to DD/ID, patient 1 had mild dysmorphic facial features, but cardiac malformations were not observed, which is consistent with previous reports on variable penetrance of cardiac malformations (Adegbola et al., 2015; Cafiero et al., 2015).

For patient 7, a novel *de novo* heterozygous variant c.1117del of *SCN2A* gene was identified, leading to a frameshift and a premature termination (p.Ala373Profs*9). *SCN2A* encodes the voltage-gated sodium channel Nav1.2, which plays a role in the initiation and conduction of action potentials (Wolff et al., 2017). Mutations in *SCN2A* were related to a spectrum of epilepsies and NDDs with phenotypic heterogeneity, including developmental and epileptic encephalopathy 11 (MIM #613721); episodic ataxia type 9 (MIM #618924); and benign familial infantile seizures 3 (MIM #607745). Patient 7 is 3 years old with DD and ID, but he is seizure-free until now. He may have later-onset epilepsy, or he may show ID and/or autism without epilepsy as 16% (32/201) of previously reported cases with *SCN2A* mutations (Wolff et al., 2017).

WES identified a novel *de novo* heterozygous nonsense mutation (c.4194T > G, p. Tyr1398*) of *ARID1B* gene in patient 8. To date, nine genes have been reported to be related to Coffin-Siris syndrome, and mutations of *ARID1B* gene were the most common reason for Coffin-Siris syndrome. *ARID1B* encodes a small subset of SWI/SNF (SWItch/Sucrose Non-Fermentable) complexes that play an important role in chromatin remodeling (Sekiguchi et al., 2019). Agenesis of corpus callosum was detected prenatally and confirmed after birth in patient 8, but hypoplasia of the fifth digits/nails was not observed, which is consistent with a previous report that most patients with corpus callosum anomalies and *ARID1B* mutations (n = 8/11) had normal fingers and toes (Mignot et al., 2016).

For patient 9, a heterozygous variant (c.649dup, p. Arg217Profs*8) of *PRRT2* gene was identified, which was inherited from his mother. *PRRT2* encodes the proline-rich transmembrane protein 2 (PRRT2) and is highly expressed in the brain and spinal cord (Chen et al., 2011). *PRRT2* is associated with familial infantile convulsions with paroxysmal choreoathetosis (MIM #602066), episodic kinesigenic dyskinesia 1 (MIM #128200), and benign familial infantile seizures 2 (MIM #605751); and the penetrance of episodic kinesigenic dyskinesia 1 is estimated to be 60%–90% (van Vliet et al., 2012). The c.649dup variant was a mutation hotspot in families of different origins (Wang et al., 2011; Ono et al., 2012; Schubert et al., 2012), and a homozygous c.649dup mutation was detected in five individuals of an Iranian family with severe non-syndromic ID (Najmabadi et al., 2011). However, it was reported that only 0.6% (8/1,423) of individuals with heterozygous *PRRT2* mutations present ID (Ebrahimi-Fakhari et al., 2015), and *PRRT2* mutations are not related to increased susceptibility to ASD (Huguet et al., 2014), suggesting that the maternally inherited c.649dup variant of *PRRT2* may not completely explain the phenotypes of ID and autism observed in patient 9.

A novel heterozygous variant c.2311dup of *SETBP1* gene was detected in patient 12, resulting in a frameshift and a premature termination (p.Ser771Phefs*26). *SETBP1* encodes the SET binding protein 1 expressed ubiquitously, but little is known about the function of *SETBP1* (Hoischen et al., 2010). *De novo* gain-of-function variants of *SETBP1* are associated with Schinzel-Giedion midface retraction syndrome (MIM #269150), while haploinsufficiency of *SETBP1* caused by loss-of-function (LoF) variant or a heterozygous gene deletion is related to DD, autosomal dominant 29 (MIM #616078). Patient 12 exhibited mild ID (IQ 65–70), consistent with a recent study that ID of various levels was observed in 77% (23/30) of patients with *SETBP1* haploinsufficiency disorder (Jansen et al., 2021). In addition, another study indicated that aberrant speech and language development are central to *SETBP1* haploinsufficiency disorder (Morgan et al., 2021), and patient 12 also showed impaired speech and language development.

For patient 14, a novel heterozygous variant c.1711del of *GRIN2B* gene was identified, leading to a frameshift and a premature stop codon (p.Ala571Profs*80). *GRIN2B* encodes the subunit NR2B of *N*-methyl-d-aspartate (NMDA) receptors, which are neurotransmitter-gated ion channels involved in the regulation of synaptic function in the central nervous system (Endele et al., 2010). *GRIN2B* is related to intellectual developmental disorder, autosomal dominant 6, with or without seizures (MIM #613970), and developmental and epileptic encephalopathy 27 (MIM #616139). A previous study of 58 individuals with *GRIN2B* encephalopathy revealed that 52% (30/58) of the patients have had seizures with a variable age of onset (0–9 years) (Platzer et al., 2017). Patient 14 is 7 years old, and he is seizure-free until now.

EIEE is a group of neurological disorders characterized by frequent epileptic seizures and development delay beginning in infancy (McTague et al., 2016). In 2018, biallelic variants in *CNPY3* gene (MIM *610774) have been identified to cause EIEE60 (MIM #617929) (Mutoh et al., 2018). *CNPY3* gene is located on chromosome 6p21.1, comprises six exons and five introns, and encodes a co-chaperone of 278 amino acid residues in the endoplasmic reticulum. In this study, two compound heterozygous variants of *CNPY3* gene were identified in patient 5. To date, three individuals with biallelic *CNPY3* variants have been reported, and their MRI showed diffuse brain atrophy and hippocampal malrotation (Mutoh et al., 2018). Brain MRI of patient 5 also showed cerebral atrophy, but hippocampal malrotation was not observed. And the other phenotypes of patient 5 were consistent with the three reported patients in the literature.

Three pathogenic CNVs were detected by exome-based CNV analysis in patients 10, 15, and 16. A 1.54-Mb heterozygous duplication on chromosome 7q11.23 was identified in patient 10; hence, he was diagnosed with chromosome 7q11.23 duplication syndrome (MIM #609757). 7q11.23 duplication syndrome is characterized by DD, speech delay, and congenital anomalies (Morris et al., 2015). To date, more than 150 individuals with 7q11.23 duplication syndrome have been reported (Mervis et al., 2015). Patient 10 was diagnosed with tetralogy of Fallot after birth and underwent surgical repair. And he showed delayed motor, speech, and autistic features at 3 years old, which are similar to the characteristics of 7q11.23 duplication patients reported in the literature.

For patient 15, a 6.46-Mb heterozygous deletion on chromosome 8p21.2p12 was detected, which overlaps with the deletion region of four cases recorded in the DECIPHER database (https://www.deciphergenomics.org). DECIPHER patient #390234 had attention deficit hyperactivity disorder, depressivity, expressive language delay, and mild ID. Patient #1557 had abnormality of the skin, blepharophimosis, fine hair, hearing impairment, hypothyroidism, ID, microtia, obesity, and short palm. Patient #415150 had mild ID, seizure. And patient #372147 had delayed speech and language development, generalized hypotonia, hypermetropia, short stature, and strabismus. A previous study reviewed 21 patients with deletions overlapping 8p21p12 region; and growth retardation, psychomotor retardation, and postnatal microcephaly were characteristics of most patients (Willemsen et al., 2009). Patient 15 also showed mild ID and DD, and he had hydrocephalus diagnosed prenatally, which was not reported in patients with deletion on 8p21.2p12. He underwent neurosurgical cerebrospinal fluid diversion after birth; afterwards, his follow-up CT scan was normal.

A 183.3-kb deletion on chromosome 19p13.2 encompassing *NFIX* gene was identified in patient 16. Haploinsufficiency of *NFIX* causes Sotos syndrome 2 (MIM #614753), which is characterized by postnatal overgrowth, macrocephaly, DD, and intellectual impairment (Malan et al., 2010). Patients with Sotos syndrome 2 will develop marfanoid habitus with age. The birth weight of patient 16 is 3.45 kg, and he had neonatal feeding difficulties. Now he is 6 years old with a weight of 21 kg (normal range) and height of 128 cm (+2 SD), consistent with a previous report that the median height of patients with Sotos syndrome 2 is 2.0 SD above the mean (range −0.5 to +3.8 SD) (Klaassens et al., 2015). He also had strabismus, DD, and moderate ID, similar to reported patients with 19p13.2 deletion encompassing *NFIX* gene (Klaassens et al., 2015; Jezela-Stanek et al., 2016).

In conclusion, WES could identify the underlying genetic causes for patients with unexplained DD/ID, and exome-based CNV analysis could detect clinically significant submicroscopic CNVs, thus improving the diagnostic yield. Our findings not only broaden the known mutation spectrum of genes associated with DD/ID but also indicate the potential of WES and exome-based CNV analysis in clinical diagnosis and discovery of disease-causing mutations and CNVs.

## Data Availability

The datasets presented in this article are not readily available due to ethical concerns regarding patient privacy and consent. Requests to access the datasets should be directed to the corresponding authors.

## References

[B1] Adegbola A. Musante L. Callewaert B. Maciel P. Hu H. Isidor B. (2015). Redefining the MED13L Syndrome. Eur. J. Hum. Genet. 23 (10), 1308–1317. 10.1038/ejhg.2015.26 25758992 PMC4592099

[B2] Brunet T. Jech R. Brugger M. Kovacs R. Alhaddad B. Leszinski G. (2021). De Novo variants in Neurodevelopmental Disorders-Experiences from a Tertiary Care center. Clin. Genet. 10.1111/cge.13946 33619735

[B3] Cafiero C. Marangi G. Orteschi D. Ali M. Asaro A. Ponzi E. (2015). Novel De Novo Heterozygous Loss-Of-Function Variants in MED13L and Further Delineation of the MED13L Haploinsufficiency Syndrome. Eur. J. Hum. Genet. 23 (11), 1499–1504. 10.1038/ejhg.2015.19 25712080 PMC4613466

[B4] Chen W.-J. Lin Y. Xiong Z.-Q. Wei W. Ni W. Tan G.-H. (2011). Exome Sequencing Identifies Truncating Mutations in PRRT2 that Cause Paroxysmal Kinesigenic Dyskinesia. Nat. Genet. 43 (12), 1252–1255. 10.1038/ng.1008 22101681

[B6] Ebrahimi-Fakhari D. Saffari A. Westenberger A. Klein C. (2015). The Evolving Spectrum ofPRRT2-Associated Paroxysmal Diseases. Brain 138 (Pt 12), 3476–3495. 10.1093/brain/awv317 26598493

[B7] Endele S. Rosenberger G. Geider K. Popp B. Tamer C. Stefanova I. (2010). Mutations in GRIN2A and GRIN2B Encoding Regulatory Subunits of NMDA Receptors Cause Variable Neurodevelopmental Phenotypes. Nat. Genet. 42 (11), 1021–1026. 10.1038/ng.677 20890276

[B9] Hiraide T. Yamoto K. Masunaga Y. Asahina M. Endoh Y. Ohkubo Y. (2021). Genetic and Phenotypic Analysis of 101 Patients with Developmental Delay or Intellectual Disability Using Whole‐exome Sequencing. Clin. Genet. 100, 40–50. 10.1111/cge.13951 33644862

[B10] Hoischen A. van Bon B. W. M. Gilissen C. Arts P. van Lier B. Steehouwer M. (2010). De Novo mutations of SETBP1 Cause Schinzel-Giedion Syndrome. Nat. Genet. 42 (6), 483–485. 10.1038/ng.581 20436468

[B11] Huguet G. Nava C. Lemière N. Patin E. Laval G. Ey E. (2014). Heterogeneous Pattern of Selective Pressure for PRRT2 in Human Populations, but No Association with Autism Spectrum Disorders. PLoS One 9 (3), e88600. 10.1371/journal.pone.0088600 24594579 PMC3940422

[B12] Ismail F. Y. Shapiro B. K. (2019). What Are Neurodevelopmental Disorders. Curr. Opin. Neurol. 32 (4), 611–616. 10.1097/WCO.0000000000000710 31116115

[B13] Jansen N. A. Braden R. O. Srivastava S. Otness E. F. Lesca G. Rossi M. (2021). Clinical Delineation of SETBP1 Haploinsufficiency Disorder. Eur. J. Hum. Genet. 29, 1198–1205. 10.1038/s41431-021-00888-9 33867525 PMC8385049

[B14] Jezela-Stanek A. Kucharczyk M. Falana K. Jurkiewicz D. Mlynek M. Wicher D. (2016). Malan Syndrome (Sotos Syndrome 2) in Two Patients with 19p13.2 Deletion Encompassing NFIX Gene and Novel NFIX Sequence Variant. Biomed. Pap. 160 (1), 161–167. 10.5507/bp.2016.006 26927468

[B15] Kearney H. M. Thorland E. C. Brown K. K. Quintero-Rivera F. South S. T. (2011). Working Group of the American College of Medical Genetics Laboratory Quality Assurance, C. (American College of Medical Genetics Standards and Guidelines for Interpretation and Reporting of Postnatal Constitutional Copy Number Variants. Genet. Med. 13 (7), 680–685. 10.1097/GIM.0b013e3182217a3a 21681106

[B16] Klaassens M. Morrogh D. Rosser E. M. Jaffer F. Vreeburg M. Bok L. A. (2015). Malan Syndrome: Sotos-like Overgrowth with De Novo NFIX Sequence Variants and Deletions in Six New Patients and a Review of the Literature. Eur. J. Hum. Genet. 23 (5), 610–615. 10.1038/ejhg.2014.162 25118028 PMC4402637

[B17] Malan V. Rajan D. Thomas S. Shaw A. C. Louis Dit Picard H. Layet V. (2010). Distinct Effects of Allelic NFIX Mutations on Nonsense-Mediated mRNA Decay Engender Either a Sotos-like or a Marshall-Smith Syndrome. Am. J. Hum. Genet. 87 (2), 189–198. 10.1016/j.ajhg.2010.07.001 20673863 PMC2917711

[B18] Manning M. Hudgins L. Professional P. Guidelines C. (2010). Array-based Technology and Recommendations for Utilization in Medical Genetics Practice for Detection of Chromosomal Abnormalities. Genet. Med. 12 (11), 742–745. 10.1097/GIM.0b013e3181f8baad 20962661 PMC3111046

[B19] Marchuk D. S. Crooks K. Strande N. Kaiser-Rogers K. Milko L. V. Brandt A. (2018). Increasing the Diagnostic Yield of Exome Sequencing by Copy Number Variant Analysis. PLoS One 13 (12), e0209185. 10.1371/journal.pone.0209185 30557390 PMC6296659

[B20] McTague A. Howell K. B. Cross J. H. Kurian M. A. Scheffer I. E. (2016). The Genetic Landscape of the Epileptic Encephalopathies of Infancy and Childhood. Lancet Neurol. 15 (3), 304–316. 10.1016/S1474-4422(15)00250-1 26597089

[B21] Mervis C. B. Morris C. A. Klein-Tasman B. P. Velleman S. L. Osborne L. R. (2015). “7q11.23 Duplication Syndrome,” in GeneReviews®. Editors Adam M. P. Ardinger H. H. Pagon R. A. Wallace S. E. Bean L. J. H. Mirzaa G. (Seattle, WA: University of Washington, Seattle), 1993–2021. Available at: https://www.ncbi.nlm.nih.gov/books/NBK327268/ 26610320

[B22] Mignot C. Moutard M.-L. Rastetter A. Boutaud L. Heide S. Billette T. (2016). ARID1Bmutations Are the Major Genetic Cause of Corpus Callosum Anomalies in Patients with Intellectual Disability. Brain 139 (11), e64. 10.1093/brain/aww181 27474218

[B23] Miller D. T. Adam M. P. Aradhya S. Biesecker L. G. Brothman A. R. Carter N. P. (2010). Consensus Statement: Chromosomal Microarray Is a First-Tier Clinical Diagnostic Test for Individuals with Developmental Disabilities or Congenital Anomalies. Am. J. Hum. Genet. 86 (5), 749–764. 10.1016/j.ajhg.2010.04.006 20466091 PMC2869000

[B24] Mithyantha R. Kneen R. McCann E. Gladstone M. (2017). Current Evidence-Based Recommendations on Investigating Children with Global Developmental Delay. Arch. Dis. Child. 102 (11), 1071–1076. 10.1136/archdischild-2016-311271 29054862 PMC5738593

[B25] Monroe G. R. Frederix G. W. Savelberg S. M. C. de Vries T. I. Duran K. J. van der Smagt J. J. (2016). Effectiveness of Whole-Exome Sequencing and Costs of the Traditional Diagnostic Trajectory in Children with Intellectual Disability. Genet. Med. 18 (9), 949–956. 10.1038/gim.2015.200 26845106

[B26] Morgan A. Braden R. Wong M. M. K. Colin E. Amor D. Liégeois F. (2021). Speech and Language Deficits Are central to SETBP1 Haploinsufficiency Disorder. Eur. J. Hum. Genet. 29, 1216–1225. 10.1038/s41431-021-00894-x 33907317 PMC8384874

[B27] Morris C. A. Mervis C. B. Paciorkowski A. P. Abdul-Rahman O. Dugan S. L. Rope A. F. (2015). 7q11.23 Duplication Syndrome: Physical Characteristics and Natural History. Am. J. Med. Genet. 167 (12), 2916–2935. 10.1002/ajmg.a.37340 PMC5005957 26333794

[B28] Mutoh H. Kato M. Akita T. Shibata T. Wakamoto H. Ikeda H. (2018). Biallelic Variants in CNPY3, Encoding an Endoplasmic Reticulum Chaperone, Cause Early-Onset Epileptic Encephalopathy. Am. J. Hum. Genet. 102 (2), 321–329. 10.1016/j.ajhg.2018.01.004 29394991 PMC5985471

[B29] Najmabadi H. Hu H. Garshasbi M. Zemojtel T. Abedini S. S. Chen W. (2011). Deep Sequencing Reveals 50 Novel Genes for Recessive Cognitive Disorders. Nature 478 (7367), 57–63. 10.1038/nature10423 21937992

[B30] Ono S. Yoshiura K.-i. Kinoshita A. Kikuchi T. Nakane Y. Kato N. (2012). Mutations in PRRT2 Responsible for Paroxysmal Kinesigenic Dyskinesias Also Cause Benign Familial Infantile Convulsions. J. Hum. Genet. 57 (5), 338–341. 10.1038/jhg.2012.23 22399141

[B31] Platzer K. Yuan H. Schütz H. Winschel A. Chen W. Hu C. (2017). GRIN2B Encephalopathy: Novel Findings on Phenotype, Variant Clustering, Functional Consequences and Treatment Aspects. J. Med. Genet. 54 (7), 460–470. 10.1136/jmedgenet-2016-104509 28377535 PMC5656050

[B32] Richards S. Aziz N. Aziz N. Bale S. Bick D. Das S. (2015). Standards and Guidelines for the Interpretation of Sequence Variants: a Joint Consensus Recommendation of the American College of Medical Genetics and Genomics and the Association for Molecular Pathology. Genet. Med. 17 (5), 405–423. 10.1038/gim.2015.30 25741868 PMC4544753

[B33] Riggs E. R. Andersen E. F. Cherry A. M. Kantarci S. Kearney H. Patel A. (2020). Technical Standards for the Interpretation and Reporting of Constitutional Copy-Number Variants: a Joint Consensus Recommendation of the American College of Medical Genetics and Genomics (ACMG) and the Clinical Genome Resource (ClinGen). Genet. Med. 22 (2), 245–257. 10.1038/s41436-019-0686-8 31690835 PMC7313390

[B34] Savatt J. M. Myers S. M. (2021). Genetic Testing in Neurodevelopmental Disorders. Front. Pediatr. 9, 526779. 10.3389/fped.2021.526779 33681094 PMC7933797

[B35] Schubert J. Paravidino R. Becker F. Berger A. Bebek N. Bianchi A. (2012). PRRT2 Mutations Are the Major Cause of Benign Familial Infantile Seizures. Hum. Mutat. 33 (10), 1439–1443. 10.1002/humu.22126 22623405

[B36] Sekiguchi F. Tsurusaki Y. Okamoto N. Teik K. W. Mizuno S. Suzumura H. (2019). Genetic Abnormalities in a Large Cohort of Coffin-Siris Syndrome Patients. J. Hum. Genet. 64 (12), 1173–1186. 10.1038/s10038-019-0667-4 31530938

[B37] Srivastava S. Love-Nichols J. A. Love-Nichols J. A. Dies K. A. Ledbetter D. H. Martin C. L. (2019). Meta-analysis and Multidisciplinary Consensus Statement: Exome Sequencing Is a First-Tier Clinical Diagnostic Test for Individuals with Neurodevelopmental Disorders. Genet. Med. 21 (11), 2413–2421. 10.1038/s41436-019-0554-6 31182824 PMC6831729

[B38] Thapar A. Cooper M. Rutter M. (2017). Neurodevelopmental Disorders. The Lancet Psychiatry 4 (4), 339–346. 10.1016/S2215-0366(16)30376-5 27979720

[B39] Utami K. H. Winata C. L. Hillmer A. M. Aksoy I. Long H. T. Liany H. (2014). Impaired Development of Neural-Crest Cell Derived Organs and Intellectual Disability Caused ByMED13LHaploinsufficiency. Hum. Mutat. 35 (11), a–n. 10.1002/humu.22636 25137640

[B40] van Vliet R. Breedveld G. de Rijk-van Andel J. Brilstra E. Verbeek N. Verschuuren-Bemelmans C. (2012). PRRT2 Phenotypes and Penetrance of Paroxysmal Kinesigenic Dyskinesia and Infantile Convulsions. Neurology 79 (8), 777–784. 10.1212/WNL.0b013e3182661fe3 22875091

[B41] Vissers L. E. L. M. Gilissen C. Veltman J. A. (2016). Genetic Studies in Intellectual Disability and Related Disorders. Nat. Rev. Genet. 17 (1), 9–18. 10.1038/nrg3999 26503795

[B42] Wang J.-L. Cao L. Li X.-H. Hu Z.-M. Li J.-D. Zhang J.-G. (2011). Identification of PRRT2 as the Causative Gene of Paroxysmal Kinesigenic Dyskinesias. Brain 134 (Pt 12), 3493–3501. 10.1093/brain/awr289 22120146 PMC3235563

[B43] Wang K. Li M. Hakonarson H. (2010). ANNOVAR: Functional Annotation of Genetic Variants from High-Throughput Sequencing Data. Nucleic Acids Res. 38 (16), e164. 10.1093/nar/gkq603 20601685 PMC2938201

[B44] Willemsen M. H. de Leeuw N. Pfundt R. de Vries B. B. A. Kleefstra T. (2009). Clinical and Molecular Characterization of Two Patients with a 6.75Mb Overlapping Deletion in 8p12p21 with Two Candidate Loci for Congenital Heart Defects. Eur. J. Med. Genet. 52 (2-3), 134–139. 10.1016/j.ejmg.2009.03.003 19303465

[B45] Wolff M. Johannesen K. M. Hedrich U. B. S. Masnada S. Rubboli G. Gardella E. (2017). Genetic and Phenotypic Heterogeneity Suggest Therapeutic Implications in SCN2A-Related Disorders. Brain 140 (5), 1316–1336. 10.1093/brain/awx054 28379373

[B46] Zhai Y. Zhang Z. Shi P. Martin D. M. Kong X. (2021). Incorporation of Exome‐based CNV Analysis Makes trio‐WES a More Powerful Tool for Clinical Diagnosis in Neurodevelopmental Disorders: A Retrospective Study. Hum. Mutat. 42, 990–1004. 10.1002/humu.24222 34015165

