# Supplementary material for: Genetic Analysis of Children With Unexplained Developmental Delay and/or Intellectual Disability by Whole-Exome Sequencing
Source: Front Genet. 2021 Nov 10;12:738561. doi: 10.3389/fgene.2021.738561 (PMC8631448; doi:10.3389/fgene.2021.738561)
Supplement: Supplementary file 1 [file DataSheet2.docx]

Supplementary table 2.Primer sequences for Sanger sequencing

| P1 | MED13L-F | TTTTCATTGGGGTGTATTGTG |
| --- | --- | --- |
|  | MED13L-R | GATGATGGTCCTGCACTGAA |
| P5 | CNPY3-Exon3-F | GTCTCTCTGGTCTGTGTTCCA |
|  | CNPY3-Exon3-R | GAGGAGCACAAACGGCAATC |
|  | CNPY3-Exon6-F | CAGGGAGGCTGATGTCAAGC |
|  | CNPY3-Exon6-R | TCCTGGGGCTTGTGTCTTTC |
| P7 | SCN2A-F | TGACTTCCTTTCTTTCCTCTAACC |
|  | SCN2A-R | TTTTCCCAGCAGCACGTAGT |
| P8 | ARID1B-F | GACTCCAATGCAAGGTGGAAGG |
|  | ARID1B-R | GGTGAACTGTTGTGGAACGGC |
| P9 | PRRT2-F | GACCCATGCCAAGAAACAGT |
|  | PRRT2-R | GGATCCATGCAGAGAGGAGA |
| P12 | SETBP1-F | GCAGGAAGCCAAGAGCAGAG |
|  | SETBP1-R | ACAGGGGACAGCGTGATTT |
| P14 | GRIN2B-F | ACGGGCATGAAGCTGTCCTTCT |
|  | GRIN2B-R | CTTGGGAGTGAAACTAGGCTTGG |
