# Supplementary material for: Genetic Analysis of Children With Unexplained Developmental Delay and/or Intellectual Disability by Whole-Exome Sequencing
Source: Front Genet. 2021 Nov 10;12:738561. doi: 10.3389/fgene.2021.738561 (PMC8631448; doi:10.3389/fgene.2021.738561)
Supplement: Supplementary file 2 [file DataSheet1.docx]

Supplementary table 1. The clinical details of 17 patients.

| **Patient ID/Sex/Age** | **Phenotype** |
| --- | --- |
| P1/Male/2y | Global developmental delay, intellectual disability, mild dysmorphic facial features. |
| P2/Male/8y | Perinatal polyhydramnios, intellectual disability, global developmental delay (delayed speech and language development, delayed fine motor development, learning disabilities), ataxia, anemia. |
| P3/Male/5y | Small head circumference was observed by ultrasound scan during pregnancy. He was born full term by natural delivery with an Apgar score of 9. He was diagnosed with spastic cerebral palsy at the age of 1 year. Febrile seizure occurred before the age of 3 and a half, but he did not have febrile seizure in the past two years. He can walk at the age of two and a half. He can call his father and mother at the age of two and a half. He can speak other words slowly at five years old, but he doesn't speak very clearly. He has made progress every year without retrogression. His mother had a history of hypothyroidism for more than 10 years, and the indicators were not well controlled during pregnancy. But he does not have hypothyroidism. The muscle tension of limbs is high, and he can't run or jump. His head circumference is small and intellectual disability is mild. |
| P4/Male/6y | Oligohydramnios was detected in the third trimester of pregnancy, and possible hypoxia was suggested. He was born by cesarean section at 36 weeks of gestation with a birth weight of 2300g. Neonatal ultrasound examination showed mild stenosis of pulmonary valva. His bilateral dislocation of hip joints were treated by operations. Clubfoot is under correction. He was diagnosed with mental retardation at the age of 3. MRI: the left ventricle was slightly larger. EEG: the energy of θ band increases at both poles. Strabismus, normal hearing, global developmental delay (delayed speech and language development, delayed fine motor development). |
| P5/Male/6y | He was born full term by natural delivery without abnormalities, weighing 2900g. He was bed ridden and presented with global developmental delay, spastic quadriplegia, intellectual disability and intractable seizure. He had multiple admissions for pneumonia and seizure. His first seizure started at age 6 months, and his EEG at 2 years of age showed diffuse sharp waves, spike waves, and multiple spike and slow wave complex. MRI of the brain at 6 year’s old revealed enlargement of lateral ventricles and cerebral atrophy. |
| P6/Female/9y | She often had fever and diarrhea before the age of 3. Drooling, dental caries, hypermetropia, intellectual disability, and global developmental delay (delayed speech and language development, delayed fine motor development). |
| P7/Male/3y | He could not sit alone six months after birth. Global developmental delay (delayed speech and language development, delayed gross and fine motor development), intellectual disability. |
| P8/Male/4y | Agenesis of corpus callosum was detected prenatally at 32 weeks of gestation, and MRI of the brain confirmed agenesis of the corpus callosum after birth. Global developmental delay (delayed speech and language development, delayed gross and fine motor development), low anterior hairline, uneven hair lines, depression between forehead and temporal bone, irregular head shape, binocular strabismus, abnormal vision, low crying. |
| P9/Male/6y | Epilepsy occurred at four month after birth. Autism was diagnosed at 2 years old. Now he is 6 years and a half, his intellectual age was 28 months. MRI shows cerebral white matter atrophy. |
| P10/Female/5y | She was diagnosed with tetralogy of Fallot after birth, and underwent surgical repair. Global developmental delay (delayed speech and language development, delayed gross and fine motor development), autism edge, abnormal development of left optic nerve and muscle, uncoordinated eyes, but normal vision. |
| P11/Male/9y | He was born full term by natural delivery with a birth weight of 3.5kg. He was prone to have cold and fever at the age of 2, and could only say "Dad" and "Mom" at the age of 3 and a half. EEG at the age of 3 showed that the left anterior temporal wave was thickened. Intelligence assessment at the age of 7 revealed an intellectual quotient (IQ) of 40 and an intellectual age of 3 and 11 months. He has the tendency of excessive diet and he is fat. Poor understanding, poor fine movements, poor coordination of movements, delayed speech and language development. His small fingers on both hands bend inward. He likes to squeak foreign bodies, has large emotional fluctuations and is easy to get excited. |
| P12/Male/7y | He was born after premature rupture of fetal membranes for several hours, with a birth weight of 2900g. Poor sucking after birth, and slow weight growth. Mild intellectual disability (IQ 65-70), delayed speech and language development, small jaw, slightly long philtrum, low-set ears, downslanting palpebral fissures, short penis. |
| P13/Male/2y | He was born prematurely at 36 weeks of gestation by cesarean section, with a birth weight of 2950g and an Apgar score of 10. He began unconscious pronunciation at 8 months, and had normal hearing. Now he is 2 years old, shows delayed speech and language development that he could only speak three to four words. |
| P14/Male/7y | He was born full term by natural delivery with a birth weight of 2900g. He had small head circumference, and had feeding difficulties after 6 months. Cerebellar hernia was observed at the age of 2, but it is not treated and under regular observation. Delayed language and motor development, normal gross movements, poor fine movements, normal language pronunciation, normal hearing, poor understanding and learning ability, hyperactivity. |
| P15/Male/7y | He was born by cesarean section at 38^+4^ weeks of gestation with an Apgar score of 10, and ultrasound showed that both lateral ventricles were 18mm. After birth, he underwent neurosurgical cerebrospinal fluid diversion, and his follow-up CT scan was normal. Echocardiography showed atrial septal defect. He can walk at the age of 2. Global developmental delay, mild intellectual disability (IQ 70), retrognathia, strabismus, slightly long philtrum, long chin. |
| P16/Male/6y | He was born by cesarean section with a birth weight of 3450g. MRI at 5 months after birth showed white matter softening. Now he is 6 years old with a weight of 21 kg (normal range), height of 128 cm (+2SD). Neonatal feeding difficulties, poor comprehension, poor ability to express long sentences, poor learning ability, moderate intellectual disability (IQ 50+), poor balance, poor fine motor movements, mild strabismus. |
| P17/Male/4y | He was severely asphyxiated at birth, and unable to sit and walk alone. Global developmental delay (delayed speech and language development, delayed gross and fine motor development), muscular hypertonia. |
